# Viral triggers of Guillain-Barré syndrome: implications for laboratory diagnostics

**DOI:** 10.11613/BM.2026.030502

**Published:** 2026-10-15

**Authors:** Karolina Kłodnicka, Jacek Januszewski, Magdalena Tyczyńska, Barbara Teresińska, Paulina Trzaskacz, Alicja Forma, Jolanta Flieger, Jacek Baj

**Affiliations:** 1 Department of Correct, Clinical and Imaging Anatomy, Medical University of Lublin, Lublin, Po-land; 2 Doctoral School, Medical University of Lublin, Lublin, Poland; 3 Department of Forensic Medicine, Medical University of Lublin, Lublin, Poland; 4 Department of Analytical Chemistry, Medical University of Lublin, Lublin, Poland

**Keywords:** antibodies, antiganglioside, Guillain-Barré syndrome, laboratory diagnostics, viral in-fection

## Abstract

Guillain-Barré syndrome (GBS) is a leading cause of acquired acute neuromuscular paralysis and is an immune-mediated disorder of the peripheral nervous system (PNS). Accumulating evidence indi-cates that GBS is often preceded by infectious events that trigger autoimmune responses through mechanisms such as molecular mimicry and autoantibody production. Campylobacter jejuni remains the most common bacterial agent, but viral pathogens also contribute to the development of GBS. This review summarizes the current evidence regarding viruses as triggers of GBS, including herpes-viruses, hepatotropic viruses (A, B, C, and E), human immunodeficiency virus, Zika virus, influenza virus, varicella-zoster virus, and the coronavirus SARS-CoV-2. Emphasis is placed on laboratory test results related to viral GBS, including antiganglioside antibodies, serological markers of recent in-fection, molecular diagnostic methods, and cerebrospinal fluid analysis. From the standpoint of la-boratory medicine, both the clinical diagnosis and the distinction of GBS subtypes can be supported by the identification of recent viral infections and the detection of immunological biomarkers. How-ever, cross-reactivity, extended antibody persistence, and inadequate assay standardization frequently make it difficult to interpret laboratory test results. In patients with probable viral GBS, this review shows the diagnostic significance and limitations of routine laboratory testing. It also highlights the significance of close coordination between laboratory professionals and clinicians.

## Graphical abstract

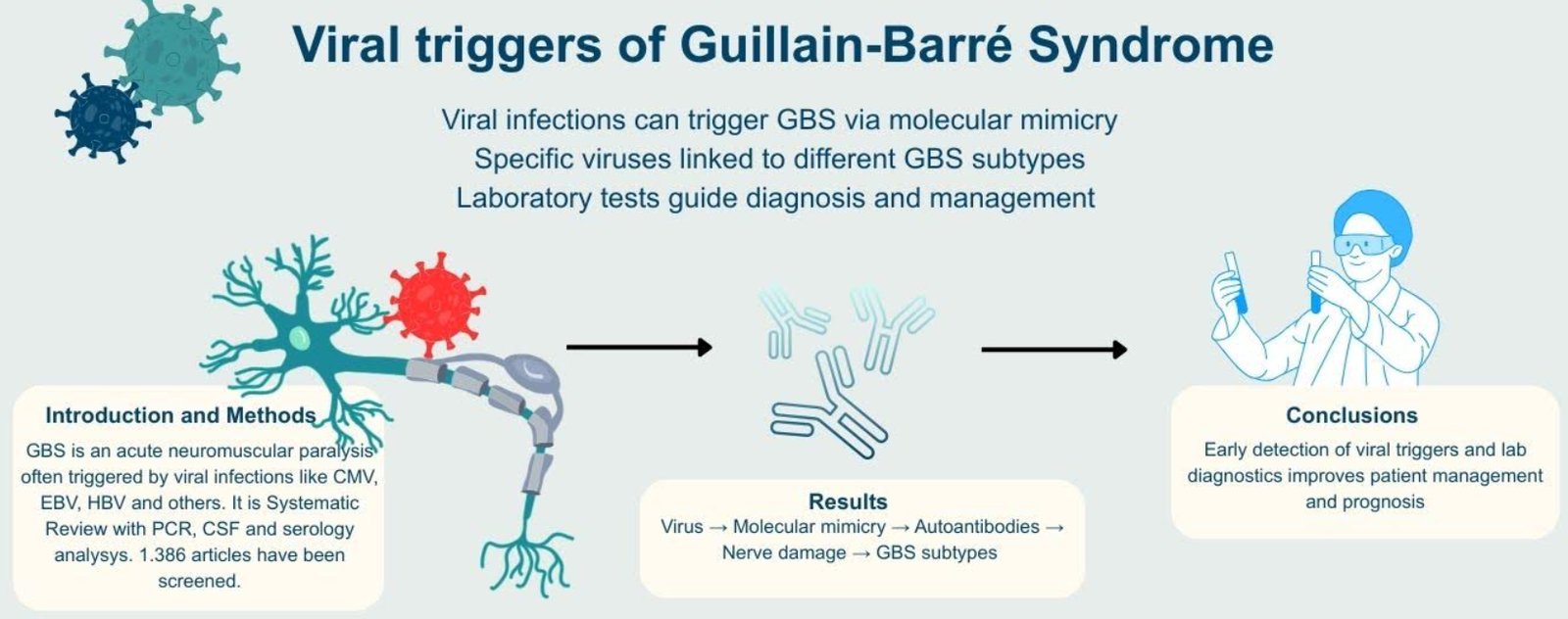

## Highlights

• Viral infections can trigger Guillain-Barré syndrome (GBS) through immune mechanisms

• Molecular mimicry between viral antigens and nerves plays a key pathogenic role

• Different viruses may induce distinct GBS subtypes with varied clinical features

• Laboratory tests support diagnosis by detecting antibodies and viral markers

• Early recognition of viral triggers improves management and patient outcomes

## Introduction

Guillain-Barré syndrome (GBS) is a postinfectious, monophasic polyradiculoneuropathy caused by immune-mediated damage to the peripheral nervous system (PNS) (*1*). It is the leading cause of ac-quired neuromuscular paralysis, often preceded by infectious events such as Campylobacter jejuni, cytomegalovirus (CMV), Epstein-Barr virus (EBV), and, more recently, severe acute respiratory syndrome coronavirus 2 (SARS-CoV-2) (*2*, *3*). While bacterial triggers are well characterized, the contribution of viral pathogens to GBS pathogenesis remains incompletely understood, representing a critical gap in both clinical and laboratory diagnostics.

Guillain-Barré syndrome manifests in different subtypes, including primary demyelinating and ax-onal variants, the latter comprising acute motor axonal neuropathy (AMAN) and acute motor and sensory axonal neuropathy (AMSAN) (*4*). Miller-Fisher syndrome (MFS), a rare variant of GBS, is characterized by ataxia, areflexia, and ophthalmoparesis, with anti-ganglioside GQ1b antibodies de-tected in approximately 85% of patients (*5*). These features highlight the immunological heterogene-ity of GBS and the potential role of virus-induced autoimmunity.

The main objective of this review is to summarize current evidence on viral triggers of GBS, with particular emphasis on their implications for laboratory diagnostics. We hypothesize that identifying viral infections and associated immunological biomarkers can improve subtype differentiation and clinical management. By providing an updated overview of viral contributions to GBS pathogenesis, this review aims to support laboratory professionals and clinicians in interpreting serological, mo-lecular, and cerebrospinal fluid (CSF) findings in suspected viral GBS cases. In order to clearly pre-sent the complexity of the phenomena discussed, a synthetic pathophysiological model illustrating the sequence of events leading from viral infection to damage to the structures of the peripheral nerve (PN) is presented in Figure 1.

**Figure 1:**
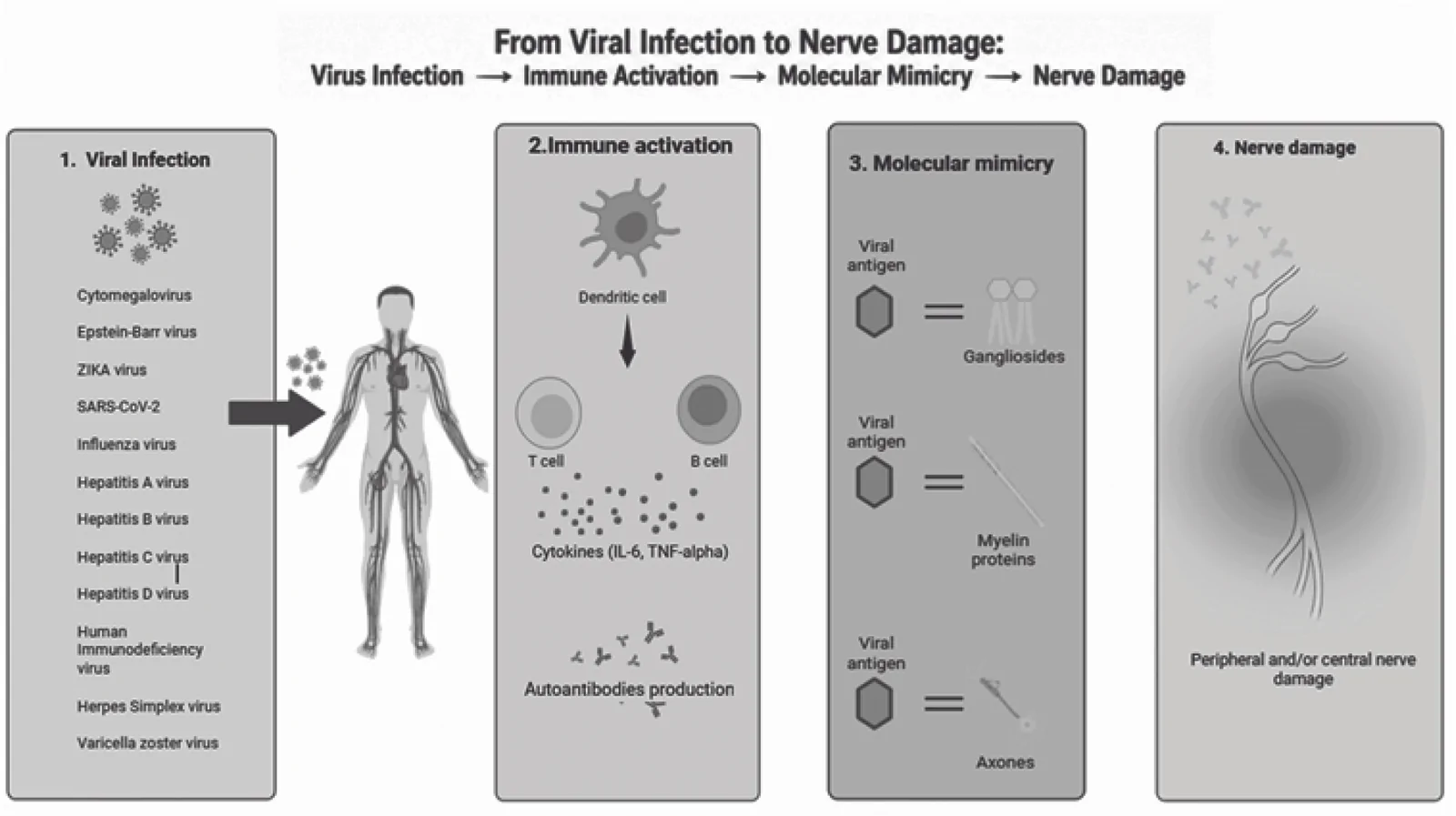
Pathomechanism of virus-associated Guillain-Barré syndrome. The figure depicts the cas-cade from viral infection to immune activation, subsequent production of autoantibodies via molec-ular mimicry, and resulting peripheral nerve damage. TNF-α - tumor necrosis factor-alpha. IL-6 - interleukin-6.

## Materials and methods

A comprehensive review of the available literature regarding viral pathogens and GBS was conduct-ed. The literature search was performed across PubMed, Google Scholar, and Scopus databases in March 2025 by two independent reviewers. Both reviewers conducted the search strategy concur-rently. The initial broad search utilized the terms: (“Guillain-Barré Syndrome” OR “Guillain Barre Syndrome”) AND “virus”. To ensure exhaustive coverage of specific pathogens, a supplementary targeted search was performed within the same timeframe using the terms: (“Guillain Barre Syn-drome” OR “Guillain-Barré Syndrome”) AND (“CMV” OR “Epstein-Barr virus” OR “Zika virus” OR “SARS-CoV-2” OR “Influenza virus” OR “Hepatitis A” OR “Hepatitis B” OR “Hepatitis C” OR “Hepatitis E” OR “HIV” OR “Herpes simplex virus” OR “Varicella zoster virus”). The search pri-marily targeted English-language articles published within the last 5 years, encompassing both hu-man and animal studies. However, seminal papers, highly relevant historical case reports, and foun-dational clinical guidelines published prior to this period were also included to provide essential context and mechanistic insights. Inclusion criteria comprised a confirmed viral infection preceding or associated with GBS and the availability of sufficient clinical or laboratory data. Exclusion crite-ria included duplicate reports across databases, off-topic studies, and articles lacking specific rele-vant information. The initial broad search yielded 1386 previously published articles. Following the removal of duplicates and a rigorous screening of titles, abstracts, and full texts against the prede-fined criteria, irrelevant and redundant studies were progressively excluded. Ultimately, 95 articles met all criteria and were included in the final review.

### Herpes simplex virus

This herpesvirus primarily affects the skin and mucous membranes and may lead to a variety of dis-eases, such as cold sores, genital herpes, herpes stromal keratitis, meningitis, and encephalitis. Her-pes simplex virus type 1 (HSV-1) is a neurotropic virus that causes a life-long latent infection in sensory ganglia following an initial epithelial infection (*6*). The association between MFS and an underlying HSV-1 infection has been recognized in a reported case of a 48-year-old man who devel-oped diplopia, bilateral ptosis, and unstable gait after an acute diarrheal illness and recurring cold sores. While the patient presented with a history of a preceding acute Campylobacter jejuni infection, the recurrent nature of the HSV-1 infection was identified as the primary precipitant for the MFS episode. The diagnosis was supported by the presence of anti-GQ1b ganglioside IgG antibodies and abnormal magnetic resonance imaging (MRI) findings in the bilateral cranial nerves III and VI. This case highlights the complex interplay between multiple pathogens and autoimmunity, suggesting that HSV-1 may provoke the formation of cross-reactive autoantibodies toward gangliosides via molecu-lar mimicry, even in the setting of prior gastrointestinal illness (*7*). This abnormal immune activity causes the development of antibodies that cross-react with ganglioside components of PNs, leading to demyelination and axonal damage typical of GBS and MFS (*8*). Another reported case involves an 80-year-old male who, after an upper respiratory tract infection, developed dysarthria, dysphonia, ophthalmoplegia, areflexia, and unstable posture. Herpes simplex virus type 1 DNA was detected in CSF analysis (*9*). This highlights the importance of detecting HSV reactivation in CSF to guide rou-tine laboratory diagnostics and combined antiviral and immunomodulatory treatment. Cross-reactivity between human herpesviruses has been suggested in previous studies (*10*). Although HSV-1 has been proposed as a potential trigger or exacerbating factor in GBS/MFS, the available evidence is limited to case reports, and no direct mechanistic link, including molecular mimicry, has been conclusively demonstrated (*11*). It is worth noting, however, that GBS as a consequence of HSV-1 infection is uncommon, with most HSV infections not leading to autoimmune neuropathy. In conclusion, HSV-1 infection can trigger GBS/MFS through complex immunological processes such as molecular mimicry and immune modulation. Early detection via laboratory testing and prompt initiation of combined antiviral and immunotherapy can improve patient outcomes (*12*).

### Varicella Zoster virus

Varicella Zoster virus (VZV) is a common human-restricted pathogen. After primary varicella infec-tion, VZV enters latency in the sensory ganglia and can later reactivate to cause herpes zoster (HZ), a disease with significant morbidity but low mortality. Both varicella and zoster are generally self-limiting, but in immunocompromised patients, they may cause serious complications, including encephalitis, myelitis, and in rare cases GBS (*13*). Herpes zoster typically presents as a localized ve-sicular rash along a dermatome and may be accompanied by sensory and motor deficits. Current evi-dence suggests that VZV can trigger GBS through molecular mimicry, where viral antigens such as glycoproteins gB, gH, and gL structurally resemble PN gangliosides (*8*). This molecular similarity may provoke an autoimmune response, leading to antibody production against host neurons. Proin-flammatory cytokines induced by the infection, particularly tumor necrosis factor-alpha (TNF-α) and interleukin-6 (IL-6), may weaken the blood-nerve barrier and facilitate leukocyte infiltration into PNs (*12*, *14*). Although GBS after VZV infection is uncommon, HZ should be considered a potential trigger. A systematic review identified 29 cases of GBS following primary VZV infection, with a median age of 37 years and a male predominance. Most patients exhibited sensory-motor deficits, including tetraparesis and cranial nerve involvement, especially the facial nerve. Intravenous immu-noglobulin (IVIG) was used as first-line therapy in all cases, with favorable outcomes in most pa-tients (*14*). There are also reports of axonal GBS variants, such as, following zoster AMAN infection, highlighting immune response heterogeneity and the possibility of seronegative presentations. In conclusion, GBS following varicella or zoster can lead to severe illness, particularly in adults, and is often associated with cranial nerve involvement (*15*). Early recognition and prompt immunotherapy are essential to improve clinical outcomes.

### Cytomegalovirus

Cytomegalovirus (CMV), a member of the Herpesviridae family, is a common viral trigger of GBS, especially in adults (*16*). CMV-associated GBS is often characterized by the presence of IgM an-ti-ganglioside antibodies, particularly anti-GM2, indicating a virus-specific immune mechanism. Laboratory confirmation typically includes CMV IgM serology, detection of viral DNA by polymer-ase chain reaction (PCR) in blood or CSF, and antiganglioside antibody assays, which can guide di-agnosis and prognosis (*17*).

For example, a 9-year-old patient developed axonal sensory-motor GBS after CMV infection, con-firmed by positive anti-CMV IgM and elevated antiganglioside antibodies. Follow-up revealed com-plete neurological recovery (*17*). Epidemiological data suggest that CMV-GBS more frequently af-fects younger individuals, with sensory deficits and facial palsy. The underlying mechanism involves molecular mimicry, where CMV antigens resemble host gangliosides, triggering cross-reactive hu-moral responses (*18*, *19*). Although CMV infection alone does not always result in GBS, immune dysregulation in susceptible hosts promotes PN injury via both humoral (anti-ganglioside antibodies) and cellular (CD8+ T-cell mediated) pathways (*19*). In summary, CMV can precipitate GBS through virus-specific immune mechanisms, and routine laboratory testing serology, PCR, and antibody de-tection plays a pivotal role in diagnosis, subtype classification, and monitoring recovery (*16*-*21*).

### Epstein-Barr virus

Epstein-Barr virus is a cause of infectious mononucleosis and has been implicated as a rare but probable trigger of GBS (*22*-*24*). Although EBV associated with GBS is uncommon, serological and immunological evidence supports a potential link. Cytomegalovirus and Campylobacter jejuni are better known as triggers of GBS because of well-defined molecular mimicry mechanisms. Ep-stein-Barr virus can cause GBS via alternative immune pathways. Proposed mechanisms include EBV-induced polyclonal B-cell activation and bystander PN components. Laboratory identification of EBV as a trigger is well established in clinical practice, including confirmed primary infection in immunocompetent patients and EBV-associated manifestations in immunocompromised individuals, such as kidney transplant recipients. Detection of EBV-specific antibodies may support the diagnosis in suspected post-infectious GBS cases, although clinical presentations may vary (*22*-*24*).

### SARS-CoV-2

The 2019 pandemic was caused by the novel coronavirus SARS-CoV-2, responsible for COVID-19, primarily affecting the respiratory system (*25*). Early reports indicated neurological involvement, both central and peripheral, including anosmia, ageusia, headache, fever, fatigue and myalgia (*26*). SARS-CoV-2 can reach the nervous system via the olfactory nerve, transsynaptic transfer, leukocyte migration across the blood-brain barier, or direct endothelial infection (*27*). Mechanisms of neural injury include direct neurotropism, immune-mediated damage, cytokine storm, coagulopathy, and molecular mimicry between viral antigens and PN components (*28*, *29*). Guillain-Barré syndrome and variants such as MFS have been reported following COVID-19 infection. A systematic review of 23 COVID-19-associated GBS cases in 2021 showed a median age of 61 years, equal gender distribu-tion, and predominance of the acute inflammatory demyelinating polyradioculopathy (AIDP) subtype over AMSAN (*30*). Severe demyelination and ICU admission were more common in these patients, highlighting the clinical significance of even rare postinfectious neuropathies (*26*, *29*). Epidemiolog-ical data also indicate an increase in GBS incidence during the pandemic compared with the previous 30 years (*31*).

### Zika virus

There are several reports describing the Zika virus (ZIKV) as a potential trigger for GBS. Detection of ZIKV RNA in serum, CSF, urine, and saliva by RT-PCR confirms viral infection and supports la-boratory diagnosis (*32*). Epidemiological studies in Colombia and Puerto Rico reported a rise in GBS cases during ZIKV outbreaks, with most patients showing neurological symptoms within four weeks after ZIKV infection (*33*). Laboratory analyses also revealed anti-glycolipid antibodies in some pa-tients, indicating a potential mechanism of molecular mimicry between viral antigens and neuronal components, especially in the AMAN subtype of GBS (*34*). Routine laboratory tests, including RT-PCR and serological assays, are essential for confirming ZIKV infection and monitoring immu-nological responses in suspected GBS cases. These findings highlight the role of laboratory diagnos-tics in identifying ZIKV-associated neurological complications (*32*-*34*).

### Influenza virus

Most epidemiological data show only occasional cases of GBS linked to influenza infection or vac-cination, though some studies suggest possible correlations (*35*). Molecular mimicry is the most widely accepted mechanism, in which gangliosides on peripheral neurons structurally resemble in-fluenza virus antigens (*36*). Immune activation during infection can generate cross-reactive antibod-ies targeting neural structures, leading to axonal damage or demyelination (*37*, *38*). During the 2009-2011 H1N1 pandemic in Norway, influenza infection but not vaccination was associated with an increased risk of GBS (*39*). Comprehensive laboratory diagnostics, including serological and im-munological assays, are essential for identifying influenza infection and monitoring immune re-sponses in patients with suspected GBS (*40*).

### Hepatitis A virus

Hepatitis A virus (HAV) is a non-cytopathic RNA virus transmitted via the fecal-oral route, causing acute hepatitis of varying severity (*41*-*45*). Although HAV is not neurotropic, several cases of GBS have been reported in temporal association with HAV infection (*46*, *47*). Molecular mimicry, where HAV antigens share epitopes with gangliosides or other PN components, is proposed to trigger cross-reactive antibodies leading to demyelination or axonal injury (*5*, *47*, *48*). Guillain-Barré syn-drome typically develops within six weeks of infection, often involving facial palsy and axonal vari-ants such as AMAN (*49*-*53*). Routine laboratory testing, including serology and immunological as-says, is essential to confirm HAV infection and to monitor immune responses in suspected GBS cas-es (*46*-*48*, *54*).

### Hepatitis B virus

Hepatitis B virus (HBV) is a hepatotropic DNA virus transmitted vertically or via contaminated body fluids (*55*). Although primarily affecting the liver, HBV infection has been rarely associated with GBS and other immune-mediated neuropathies (*56*-*59*). Mechanisms include molecular mimicry, immune complex formation, and T-cell dysregulation, which may lead to production of cross-reactive antibodies against PNs (*60*–*63*). Immune complexes containing HBsAg have been de-tected in both serum and CSF of the HBV infection-associated GBS case, supporting laborato-ry-based diagnosis and monitoring of immune-mediated damage (*59*-*63*). Awareness of this rare as-sociation is important, as early interventions such as IVIG or plasma exchange (PLEX) can improve neurological outcomes (*64*).

### Hepatitis C virus

Hepatitis C virus (HCV) chronically infects approximately 58 million people worldwide and is transmitted via blood and high-risk sexual contact (*65*, *66*). Hepatitis C virus exerts immunomodula-tory effects that can predispose to autoimmune complications, including GBS (*66*, *67*). Immuno-pathogenic mechanisms involve persistent activation of innate and adaptive immunity, B-cell-driven autoimmunity, molecular mimicry, and immune complex deposition (*68*-*70*). Deposition of immune complexes in small vessels supplying PNs may result in vascular and demyelinating neuropathies. Laboratory tests, including serology and detection of immune complexes, are critical in assessing infection status and monitor autoimmune responses in HCV-infected patients with neurological complications. Long-lasting immune activation triggered on by chronic HCV infection is character-ized by persistent inflammatory signals and changes in humoral and cellular immune responses. Furthermore, persistent immunological abnormalities such as continuous macrophage activation fol-lowing viral elimination, suggest that HCV may have prolonged immunomodulatory effects that may promote immune-mediated issues resulting from infection (*69*-*71*).

### Hepatitis E virus

Hepatitis E virus (HEV) infection has been linked to GBS through aberrant immune responses (*72*). Molecular mimicry, with antibodies or T cells targeting viral antigens cross-reacting with PN com-ponents such as gangliosides, contributes to inflammatory and demyelinating nerve damage (*73*-*75*). Hepatitis E virus genotype 1 is most commonly associated with neurological complications, with autoantibodies targeting nodal and paranodal proteins, disrupting nerve conduction and promoting demyelination and axonal degeneration via complement activation (*75*, *76*). Laboratory detection of HEV infection and monitoring of immunopathogenic markers are important for identifying patients at risk of HEV-associated GBS (*72*-*76*).

### Human immunodeficiency virus

Guillain-Barré syndrome has been reported in association with human immunodeficiency virus (HIV) infection, primarily in case reports since the 1980s (*77*-*79*). Guillain-Barré syndrome usually occurs early during acute retroviral syndrome or seroconversion, but can also develop during chronic HIV infection (*80*-*82*). Proposed mechanisms include autoimmune responses triggered by molecular mimicry between viral antigens and PN components, bystander activation of immune cells, and im-paired regulation of autoreactive T cells, which may lead to demyelination and axonal damage (*83*, *84*). Immune reconstitution inflammatory syndrome (IRIS) after initiation of highly active antiretro-viral therapy (HAART) may also contribute to GBS onset. Routine laboratory testing, including CSF analysis showing elevated protein concentrations and immunological assays - such as markers of intrathecal immune activation, the assessment of anti-ganglioside antibodies and CD4+ T cell counts - is essential for diagnosis and monitoring of HIV-associated GBS (*85*, *86*). CD4+ T cell depletion, chronic immune activation, and endothelial dysfunction in untreated HIV infection may further pre-dispose to autoimmune neuropathies (*87*-*92*). The British HIV Association (BHIVA) recommends HIV screening in all patients presenting with GBS (*93*).

### Laboratory diagnostics

Accurate laboratory diagnostics is critical in confirming virus-associated GBS and its variants, in-cluding MFS, and in guiding immunotherapy and antiviral interventions. Routine laboratory ap-proaches combine serological, molecular, and immunological methods to provide a comprehensive assessment of viral exposure, immune activation, and PN involvement.

In alignment with current international consensus guidelines, CSF analysis remains a cornerstone of the diagnostic workup for GBS, where a separate evaluation of biochemical and cellular parameters is required (*2*, *8*). The expected laboratory findings in patients with GBS are characterized by albu-minocytologic dissociation, defined as an elevated CSF protein concentration (> 0.45 g/L) accompa-nied by a normal CSF leukocyte count (< 10 cells/μL) or, less commonly, a mildly elevated leuko-cyte count (< 50 cells/μL) (*1*). According to current diagnostic criteria and management frameworks, this classical pattern supports the clinical diagnosis but its absence, particularly during the first week of symptom onset when protein concentrations can still be normal, does not exclude GBS (*2*).

For example, serological assays detecting virus-specific IgM and IgG antibodies are central in diag-nosing infections such as CMV, EBV, HSV-1, and hepatitis viruses, and in differentiating acute from past infections (*6*, *9*, *11*, *14*, *16*, *17*, *20*, *22*-*24*, *46*, *55*, *57*, *62*, *65*, *69*, *94*). Molecular diagnostics, par-ticularly PCR, enhance detection sensitivity by identifying viral DNA or RNA in serum, CSF, urine, or saliva. PCR-based confirmation is especially valuable in cases with atypical serological results, early infection before seroconversion, or reactivation of latent viruses such as HSV-1 or VZV (*6*-*9*, *13*). In CSF, PCR detection of viral nucleic acids, together with protein concentration assessment, cell count, and immunoglobulin indices, can clarify whether neuropathy is driven primarily by direct viral neurotropism or post-infectious autoimmune mechanisms (*9*, *85*, *86*). For instance, HSV-1 DNA detected in CSF in GBS patients supports combined antiviral and immunomodulatory treatment (*9*). Similarly, detection of Zika virus RNA by RT-PCR confirms infection and supports the identification of molecular mimicry driven AMAN subtype GBS (*32*-*34*). Laboratory testing is also essential for early detection of viral reactivation or co-infections, which may modulate the risk of autoimmune neuropathy. For example, CMV IgM and IgG positivity alongside transient EBV IgM positivity sug-gests that multiple viral exposures can contribute synergistically to disease onset (*94*). Similarly, in SARS-CoV-2-associated GBS, serological and molecular analyses can differentiate post-infectious autoimmune neuropathy from direct viral neurotropism, guiding the use of immunomodulatory ther-apy in critically ill patients (*26*, *29*, *30*).

In laboratory diagnosis, antiganglioside antibody tests play a key role. They reflect the immunologi-cal mechanisms triggered by various pathogens, including viruses and bacteria (*8*). A key aspect of the pathophysiology of GBS is the development of antiganglioside antibodies. They arise through molecular mimicry between viral or bacterial antigens and PN components (*3*, *8*, *76*). These antibod-ies are typically detected in serum using enzyme-linked immunosorbent assay (ELISA) or im-munoblot techniques (*5*, *70*). Viral infections can trigger production of antibodies against ganglioside components of PNs, including GM1, GM2, and GQ1b, through molecular mimicry (*3*, *8*, *34*). Sero-logical detection of these antibodies allows classification of GBS subtypes (AIDP, AMAN, AMSAN) and helps predict clinical outcomes. Their clinical significance lies in their clear association with specific clinical variants of GBS. Anti-GQ1b antibodies are highly specific for MFS, whereas anti-bodies against GM1 or GD1a gangliosides are often associated with axonal forms of GBS, such as AMAN (*5*, *18*, *75*). Identification of these antibody profiles is a key diagnostic tool for subtyping and prognostic assessment (*5*, *18*, *69*). Moreover, sequential measurement of antibody titers can monitor immune response dynamics and treatment efficacy during IVIG or plasma exchange therapy (*8*, *10*, *15*, *95*). In EBV-associated GBS, polyclonal B-cell activation may lead to the production of diverse autoantibodies, while detection of EBV capsid-specific antibodies may support diagnosis when con-sidered alongside clinical findings (*22*-*24*). Additional biochemical analyses, including measurement of inflammatory cytokines, chemokines, and complement components in serum and CSF, may fur-ther characterize immune activation and peripheral nerve injury (*10*, *13*, *95*). Regarding the position-ing of serological biomarkers within current diagnostic algorithms, antiganglioside antibody testing is officially recognized as a supportive, secondary diagnostic tool rather than a mandatory criterion (*2*, *17*). While their routine screening is highly recommended to narrow down specific clinical phe-notypes and differentiate GBS variants from other acute neuropathies, current management guide-lines emphasize that therapeutic interventions (such as IVIG or plasma exchange) should never be delayed while awaiting antibody test results (*2*, *5*, *17*). Interpretation of the results of these tests, however, requires careful consideration of diagnostic sensitivity and specificity, which vary dramat-ically depending on the GBS subtype (*5*). IgG anti-GQ1b antibodies demonstrate exceptional diag-nostic sensitivity (approximately 85%) and high specificity for MFS (*5*). In the case of primary de-myelinating variants, such as acute inflammatory demyelinating polyradiculopathy (AIDP), the clin-ical sensitivity of standard antiganglioside panels remains relatively low (approximately 40-60%). This means that a negative serological result cannot completely exclude the diagnosis of GBS (*5*, *69*). However, when specific IgM anti-GM2 antibodies are detected in acute CMV infection or IgG an-ti-GM1/GD1a in axonal variants, such as AMAN, their diagnostic specificity confirms the GBS sub-type and helps determine the clinical prognosis (*5*, *17*, *69*). Modern laboratory diagnostics rely on integrating serological test results with patient clinical profiles. The complex relationships between viral etiology, clinical phenotype of GBS, and antiganglioside antibody profiles require a systematic diagnostic approach.

Complementary biochemical assays enhance diagnostic accuracy. Measurement of inflammatory cytokines, chemokines, and complement components in serum and CSF can provide mechanistic in-sight into virus-induced immune activation and PN injury (*10*, *13*, *95*). Elevated proinflammatory cytokines may facilitate leukocyte infiltration across the blood-nerve barrier and exacerbate demye-lination in VZV or influenza-associated GBS (*15*, *36*). Immune complex deposition, particularly in hepatitis B and C infections, can be identified through laboratory detection of HBsAg or HCV-antigen complexes in serum and CSF, elucidating the pathway of T-cell dysregulation and an-tibody-mediated nerve injury (*59*, *69*-*71*). Furthermore, the acute inflammatory cascade triggered by viral pathogens induces robust generation of reactive oxygen species (ROS), leading to significant oxidative stress that accelerates mitochondrial dysfunction and lipid peroxidation within peripheral myelin and axonal membranes (*5*, *8*). New clinical studies highlight the significant role of structural biomarkers that reflect direct damage to PNs and axons in viral GBS (*5*, *8*, *69*). Among these, neuro-filaments, and especially tau protein, have gained importance as surrogate markers of axonal degen-eration and cytoskeletal damage resulting from intense autoimmune insults (*17*, *69*). In patients with AMAN, significantly elevated concentrations of neurofilament light chain and tau in both CSF and serum correlate with the degree of PN damage and may serve as valuable indicators of long-term clinical outcome (*2*, *5*, *69*). Similarly, changes in peripherin, an intermediate filament protein ex-pressed primarily in peripheral neurons, reflect structural remodeling and stress responses of axonal networks during acute demyelinating or axonal attacks (*7*, *17*). These structural biomarkers are now viewed as powerful research tools. Assessment of neurofilaments, peripherins, and tau protein pro-vides pathobiological information on the severity of virus-induced neurodegeneration and gives hope for the development of personalized prognostic algorithms in the future (*2*, *17*, *69*).

Finally, integrating laboratory findings into clinical decision-making is vital for optimizing patient outcomes. Combined interpretation of serology, PCR, antiganglioside antibodies, CSF biochemistry, and immune markers provides a robust framework for identifying virus-specific triggers, confirming GBS subtypes, and guiding timely interventions (*16*-*21*, *32*-*34*, *85*, *86*). Early and precise laboratory evaluation allows clinicians to anticipate potential complications, select appropriate antiviral or im-munomodulatory therapy, and monitor neurological recovery, ultimately improving prognosis and reducing long-term disability. A summary of key biomarkers, recommended analytical methods, and diagnostic limitations for individual viral pathogens is presented in Table 1.

**Table 1:** Associations between viral triggers, GBS clinical subtypes, and key anti-ganglioside anti-body profiles

**Viral trigger**	**Associated GBS subtype(s)**	**Key antiganglioside antibodies**
CMV	AIDP, AMAN, Sensory GBS	Anti-GM2 (IgM)
EBV	AIDP, MFS	Anti-GM1, Anti-GD1a
VZV	AIDP, AMAN	Anti-GM1, Anti-GD1b
HSV-1	AIDP, MFS	Anti-GD1a, Anti-GQ1b (rare)
SARS-CoV-2	AIDP, AMAN, MFS	Anti-GD1a, Anti-GM1
ZIKV	AMAN (frequent autonomic dysfunction)	Anti-GA1, Anti-GM1
Influenza virus	AMAN, AIDP	Anti-GM1, Anti-GD1a
HBV / HCV	AIDP (associated with vasculitis)	Anti-GM1 (rare)
HIV	AIDP (seroconversion or IRIS)	Anti-GM1, Anti-glycolipid complexes
CMV – Cytomegalovirus. EBV - Epstein-Barr virus. VZV - Varicella-zoster virus. HSV-1 - Herpes simplex virus type 1. SARS-CoV-2 - Severe acute respiratory syndrome coronavirus 2. ZIKV - Zika virus. HBV - Hepatitis B virus. HCV - Hepatitis C virus. HIV - Human immunodeficiency virus. AIDP - Acute inflammatory demyelinating polyneuropathy. AMAN - Acute motor axonal neuropathy. GBS - Guillain-Barré syndrome. MFS - Miller Fisher syndrome. IRIS - Immune reconstitution inflammatory syndrome. IgM - Immunoglobulin M. GM1, GM2, GD1a, GD1b, GQ1b, GA1 - Gangliosides (standard nomenclature).		

In summary, laboratory diagnostics in virus-associated GBS are multifaceted, encompassing serolo-gy, molecular detection, antibody profiling, and immunochemical analysis. These methods not only confirm viral triggers but also elucidate the biochemical and immunopathogenic mechanisms under-lying PN injury, emphasizing their central role in research and clinical management of virus-induced neuropathies.

## Discussion

The reviewed evidence suggests that viral infections can trigger GBS (*7*, *9*, *12*). However, a critical review of the current literature has shown that the clinical utility of laboratory markers remains in-consistent (*11*, *37*, *49*). Molecular mimicry is a widely accepted mechanism. A key challenge is the high heterogeneity of antibody responses. CMV-associated GBS often presents with IgM anti-GM2 antibodies, which strongly correlate with sensorimotor deficits and facial nerve paralysis, providing a reliable diagnostic link (*9*, *15*, *51*). In contrast, EBV-associated GBS lacks a single pathognomonic marker. It often demonstrates polyclonal B-cell activation, complicating differentiation from other postinfectious neuropathies (*23*, *94*). Although SARS-CoV-2 has been associated with GBS, the role of direct neurotropism remains debated. Laboratory results often demonstrate elevated protein con-centrations in CSF (*1*, *58*, *80*). Unlike C. jejuni or CMV infections, antiganglioside antibody profiles are much less predictable in COVID-19 (*67*, *90*).

Our analysis highlights the importance of identifying virus-specific markers and antiganglioside pro-files for differentiating GBS subtypes. The presence of anti-GQ1b antibodies in patients with oph-thalmoplegia and ataxia confirms the diagnosis of MFS, regardless of whether the trigger is HSV-1 or other pathogens (*2*, *5*, *12*). Axonal variants (AMAN/AMSAN) are more frequently associated with infections triggering anti-GM1 or anti-GD1a antibodies, often associated with ZIKV or CMV (*37*, *49*, *80*). However, clinical interpretation of these findings is often hampered by cross-reactivity and the transient nature of antibody titers. Future studies should focus on long-term monitoring of these bi-omarkers to determine their predictive value for treatment response (*1*, *58*, *94*). Integrating structural markers such as neurofilaments with traditional serological testing may provide a more robust framework for the treatment of patients with GBS, moving beyond a simple “positive/negative” di-agnosis towards a personalized, subtype-specific treatment strategy (*1*, *49*, *67*).

In conclusion, the primary finding of this review is that the viruses discussed can function as triggers for GBS. Impairment of the immune response, production of autoantibodies, molecular mimicry, and oxidative stress are likely mechanisms contributing to GBS onset. Several of the presented pathogens also exhibit neurotropic capabilities, facilitating direct or indirect nervous system injury. These ob-servations highlight the importance of considering recent viral infections in patients presenting with early neurological symptoms, enabling timely initiation of appropriate treatment, and potentially reducing the need for intensive care and complications such as respiratory failure. Although our un-derstanding of virus-associated GBS has advanced, many pathophysiological mechanisms remain incompletely elucidated, and optimal therapeutic strategies are still under investigation. Future re-search, including well-designed clinical studies, is essential to refine laboratory diagnostics, clarify immunopathogenic mechanisms, and improve prevention and management of GBS worldwide.

## Data Availability

No data was generated during this study, so data sharing statement is not applicable to this article.
